# On Intra-Pelvic Phlegmonous Abscesses

**Published:** 1845-04

**Authors:** 


ART. VI.—*Des Abscès Phlegmomeneux Intra-Pelviens.* Par M. MARCHAL (DE CALVI).—*Paris,* 1844.

*On Intra Pelvic Phlegmonous Abscesses.* By M. MARCHAL (DE CALVI). —*Paris,* 1844. 8vo, pp. 196.

THIS brochure is a reprint from the 'Journal de Chirurgie' of a series of articles which appeared in that publication at the commencement of last year. Its merits are entirely those of a compilation, since, though it contains the particulars of seventy-five observations, not one of the cases came under the author's own notice. It treats of these abscesses in the male as well as in the female subject, though, as might be expected, by far the greater number of cases refer to females, since the process of parturition is a cause of inflammation of the cellular tissue and soft parts within the pelvis, to which nothing analogous exists in the male sex. Fifty-two cases are recorded of phlegmonous abscess of the pelvis as a consequence of labour. Forty-four of these cases have already appeared in print; the remaining eight were communicated by M. Bouchat, a friend of the author, and formerly Interne at the Maternité. Eight other cases are related which occurred independently of labour; but when we find the author reporting under this head cases of suppuration or tuberculous degeneration of the ovaries, he appears to us to be departing from his real subject, and to be writing an exceedingly imperfect essay on the diseases of the internal organs of generation. The remaining fifteen observations refer either to the male subject, or to cases in which the sex of the patient had nothing to do with the occurrence of the affection; which depended on disease of the intestine, inflammation of the bladder, peritonitis, phlebitis, &c. &c. These cases, which as well as the others are mostly extracted from French writers, are all detailed at full length; a practice which has rendered the memoir needlessly prolix, though perhaps it adds to its value as a repository of facts which some subsequent writer may better understand how to employ.

